# Cellular systems for epithelial invagination

**DOI:** 10.1098/rstb.2015.0526

**Published:** 2017-03-27

**Authors:** Esther J. Pearl, Jingjing Li, Jeremy B. A. Green

**Affiliations:** Department of Craniofacial Development and Stem Cell Biology, King's College London, London SE1 9RT, UK

**Keywords:** epithelial bending, apical constriction, basal relaxation, basal wedging, cellular tractoring, suprabasal intercalation

## Abstract

Epithelial invagination is a fundamental module of morphogenesis that iteratively occurs to generate the architecture of many parts of a developing organism. By changing the physical properties such as the shape and/or position of a population of cells, invagination drives processes ranging from reconfiguring the entire body axis during gastrulation, to forming the primordia of the eyes, ears and multiple ducts and glands, during organogenesis. The epithelial bending required for invagination is achieved through a variety of mechanisms involving systems of cells. Here we provide an overview of the different mechanisms, some of which can work in combination, and outline the circumstances in which they apply.

This article is part of the themed issue ‘Systems morphodynamics: understanding the development of tissue hardware’.

## 1. Epithelial invagination as a multicellular mechanism

In animal development from the very earliest blastocyst or blastoderm stages all the way to the very last stages of organogenesis, embryos organize themselves into epithelial layers. Epithelium is broadly defined. It can be a sheet of cuboidal, columnar or squamous (flattened) cells, or contain a mixture of cell shapes of varying height to give the appearance of multiple layers (pseudostratification), or even consist of any of the above in multiple layers and be truly stratified. However, for all stages and all epithelial types, elaboration of anatomy relies on the self-bending ability of epithelia into folds, ridges, pits and tubes. As a building block of morphogenesis, epithelial bending makes almost every organ, from the primitive gut tube that makes the primary body axis during gastrulation to the finest pores that are the hair follicles on the skin. Epithelial bending is self-evidently a multicellular process in which multiple connected cells coordinate their behaviours to change the shape of the tissue. Put another way, epithelial bending is an emergent property of a system of cells whose actions cannot be described at lower levels: gene networks and classical (largely subcellular) cell biology cannot fully capture the epithelial bending process. Remarkably, despite its being a very widespread process, our detailed descriptions and mechanistic understanding of epithelial bending are limited to rather few cases and types.

Aspects of epithelial bending leading to both invagination (folding inwards) and evagination (folding outwards) have been reviewed previously [1–4]. This review focuses on bending that results in invagination of the epithelium, from the point of view of cellular behaviours. We start our summary from the fairly well described apical constriction, via apical cable-driven buckling, cell shortening by other mechanisms and basal wedging, to apical/basal bunching and vertical telescoping to the relatively novel and little-characterized suprabasal intercalation. This order reflects the hierarchy of epithelial complexity from a monolayer to pseudostratified, and finally stratified structure. It also reflects a hierarchy of complexity in the cellular processes involved.

## 2. Apical constriction

Apical constriction is defined as a mechanism in which epithelial cells undergo apical shrinkage while keeping a more or less constant volume [5]. Several good reviews have recently been published on apical constriction [1,4,6–10] and the reader is directed to those for a comprehensive analysis. Here we will outline some salient features.

**Figure 1.**
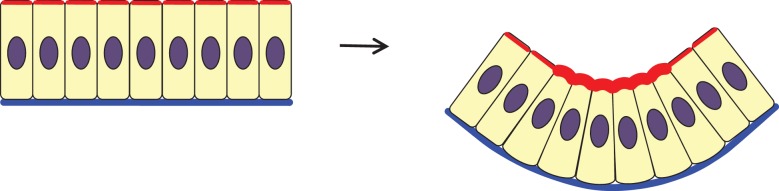
Classical apical constriction. In a monolayer where cells keep constant volumes, accumulated actomyosin meshwork at the apical end of the cells constricts, giving rise to wedge-shaped cells. This forces the epithelium into a concave apical surface with an enlarged basal area. Red, actomyosin (note enrichment on the apical side of the cells); blue, basal lamina; purple, nucleus.

Early two-dimensional physical models made with steel rods and rubber tubing demonstrated that differential tension between the apical and basal surfaces of epithelial cells would lead to bent epithelia, provided cell volume and height were maintained [11]. Additionally, early observations of epithelial bending across a range of organs and organisms showed that the cells in the bending tissue that are wedge-shaped have a superficial gel layer at the concave side of the curvature [11]. This contracting gel layer was later discovered to consist of actin filaments [12], acting in concert with the motor protein myosin II to bend the epithelium (figure 1). Apical actomyosin enrichment and contractility have become defining characteristics of apical constriction [13–16]. Regulation of the actomyosin cytoskeleton is complex, but among the numerous regulators, the recruitment of this contractile machinery is notably promoted by Rock [17,18] and Shroom [19–22]. Further studies have shown that while Shroom is both necessary and sufficient for the apical distribution of the actomyosin contractile network [19,20], other molecules very often function in positioning distinct components of the machinery to the correct place. For instance, Rho GTPase [17] and p120 catenin [13] are required to localize myosin II apically in the cell. BMP, acting upstream of Rock in chick otic placode (neuroepithelial) invagination, seems to be required for apical localization of actin independently of a role in cell type specification [23].

Live imaging of invaginating tissues has provided an increasingly sophisticated picture of how apical constriction takes place. For example, it was long assumed that cells undergo apical constriction by a purse-string-like contraction of actin fibres around the circumference of the apical surface. Live imaging in *Drosophila* gastrulation revealed that, instead of circumferential fibres, an apical meshwork of diametrical fibres actually plays the predominant role in constricting the apical area [15] (although there is currently no equivalent evidence in vertebrates). The process of contraction is also less simple than once thought. Rather than smooth and synchronous contraction, it has recently been demonstrated that individual cells undergo transient pulses of ratchet-like constriction asynchronously with their neighbours [15,16,24–26]. After contractions are initiated, the contracted state is stabilized between pulses so that the net result is a decrease in the area of the apical end of the cell [15,24]. The tension from these individual contractions is probably transmitted apicobasally by cytoplasmic displacement, at least as is seen in *Drosophila* mesoderm [27]; simultaneously, the tension is transmitted in the plane of the tissue via the actomyosin network, which is assembled in individual cells and connected intercellularly by adherens junctions [24], to bend the whole tissue.

## 3. Basal relaxation

**Figure 2.**
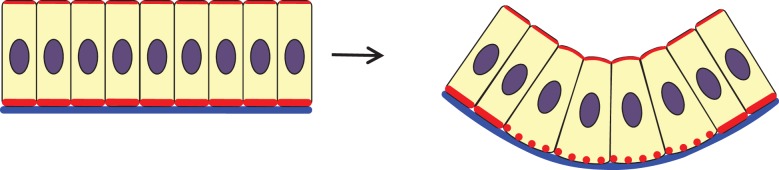
Basal relaxation. Basal relaxation is observed as a stage preceding apical constriction in some contexts. Active disassembly of F-actin at the basal end of the cells facilitates wedging of these cells as well as later apical accumulation of actomyosin cables, which subsequently deform the epithelium. Solid red lines, F-actin; dotted red curve, disassembled actin filaments on the basal side of cells; blue, basal lamina; purple, nucleus.

If cell volume is to be conserved, apical constriction must be accompanied by either basal expansion or height increase (or both). Increase in height has been observed in tracheal and salivary gland placodes before invagination in fly embryos [28,29], and what we call ‘basal relaxation’ here, in which the basal actin or myosin network is actively disassembled (figure 2), has been reported as being involved in the invagination of the chick otic placode [30–32] and *Drosophila* ventral furrow formation in gastrulation [5]. In the chick otic vesicle, basal relaxation precedes apical constriction and depends on basally presented FGF signals [30], and so does not seem to be necessarily coupled to apical events, including the subsequent constriction. In *Drosophila* gastrulation, however, reduction of basal myosin intensity and in turn basal rigidity accompanies apical constriction and expands the basal surface, a phase that very likely initiates the transition from cell columnization to cell shortening and invagination [5]. A recent paper by Lomakin *et al.* [33] has suggested that actomyosin accumulation in one part of a cell during migration causes depletion in another. This could be a way in which basal relaxation could trigger or be necessary for subsequent apical constriction during invagination. Unpublished computer modelling of epithelial folding in wing disc epithelium has suggested that basal relaxation in that context may in fact be mechanically more important than apical constriction (Guillaume Salbreux 2016, personal communication).

## 4. Apical cable-driven buckling

In a number of contexts, the contractility of multiple cells is coordinated via actomyosin ‘cables’ [34,35]. Actomyosin cables are supracellular structures contained within individual cells that align between adjacent cells [34–36] and are probably connected via specific junctions, although how they are connected at the molecular level is still unknown. These supracellular structures have been observed not only during invagination [36,37], but also in other processes [38–40], to coordinate contraction.

One example of actomyosin cable-driven invagination is chicken neural tube closure, in which mediolaterally orientated myosin cables run several cell lengths, promoting cell intercalation mediolaterally to both elongate the neural tube (convergent extension) and bend the neuroepithelium mediolaterally [37,41]. This planar-polarized contraction of actomyosin cables is promoted by upstream PCP signalling and also polarized distribution of Celsr1 and ROCK [41].

**Figure 3.**
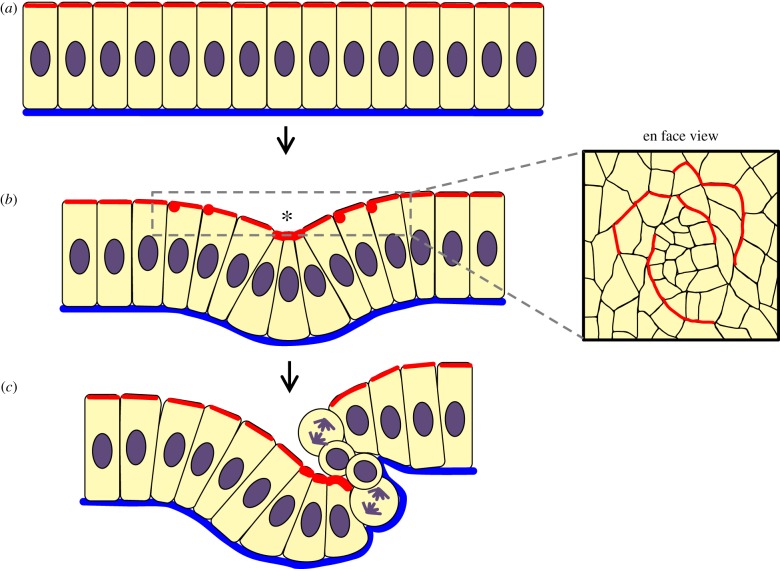
Apical cable-driven buckling, case 1. In the developing *Drosophila* tracheal pit, apical cables (actomyosin structures aligned in adjacent cells) generate constriction that bends and buckles the epithelial sheet assisted by mitotic rounding of cells. (*a*) Immediately before invagination, cells in the tracheal placode undergo a period of mitotic quiescence. (*b*) A limited number of cells at the centre of the placode then constrict apically. The contractile force of the actomyosin cable arcs further away from the centre (red dot in lateral view, cross section of cable; box to right, en face view of cable arcs) helps compress cells towards the centre of the invagination. (*c*) Invaginating cells round during mitosis, causing a rapid drop in cell height and deforming neighbours simultaneously. Red, actomyosin; blue, basal lamina; purple, nucleus/mitotic spindle; *, apical constriction; red, cross sections through actomyosin cable arcs.

The epithelium in the developing *Drosophila* uses constriction coupled with cellular rearrangement and cell rounding to achieve invagination of multiple tracheal pits, which will later form the tracheal network through which oxygen diffuses towards fly tissues [36,42]. Prior to the start of invagination, cells in the placode enter mitotic quiescence [36]. Short circumferentially aligned arcs of actomyosin cables form transiently as groups of a few cells intercalate (likewise circumferentially) around the forming pit [36]. This is followed by strong apical constriction of the cells at the very centre of the placode and less tightly constricted apices in immediately surrounding cells, forming a shallow tracheal pit [36]. The invaginating cells at the centre undergo mitotic cell rounding which accelerates the process by causing a rapid drop in cell height, finishing the invagination in a rapid phase [42]. It was shown that it is the rounding of the mitotic cells but not cell division that drives the rapid phase of invagination. One can speculate that rounded cells make the epithelium structurally weaker. They have a less stiff cortical cytoskeleton, a less columnar shape (thinning the epithelium) and possibly weaker attachments to their neighbours. They could therefore act as buckling points at which the epithelium bends with less resistance to the tension maintained by the circumferential cables in the surrounding non-dividing cells (figure 3).

## 5. Cell shortening

**Figure 4.**
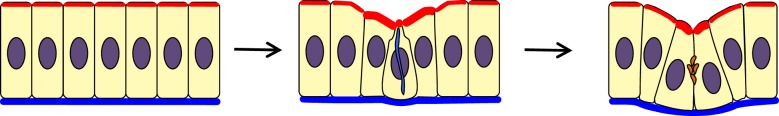
Apical cable-driven buckling, case 2. Some tissues, including folding in early *Drosophila* leg epithelium, use apoptosis to assist apical constriction. Mechanical forces that bend the epithelium in this case are thought to be produced by an apicobasally orientated actomyosin cable (pale blue) in the dying cell, which acts as a (not necessarily passive) buckling point of the invagination. Red, actomyosin; dark blue, basal lamina; pale blue line, apoptotic actomyosin cable; orange, apoptotic fragments; purple, nucleus.

Folding of the *Drosophila* leg epithelium to make joints between segments represents another variation of cellular constriction, which is in this case whole-cell shrinkage coupled with apoptosis [30,43,44]. During the morphogenesis of *Drosophila* leg epithelium, apoptosis is necessary, but not sufficient, for apical constriction to occur [45], and a relatively recent report describes an apicobasal actomyosin ‘cable’ running vertically through the centre of the cell at the folding placode (figure 4) that appears as though it might exert a downward vertical pulling force on the apical surface of the neighbouring cells [30]. These vertical ‘cables’ are not to be confused with the planar arcs of actomyosin cables referred to in §4 and are entirely novel single-cell structures whose structure and dynamics remain to be investigated. As with mitotically rounding cells, an apoptotic cell would presumably be structurally weaker than its non-apoptotic neighbours and therefore could serve as a buckling point; however, the apicobasal ‘cable’ hints at a more active mechanism, as does the fact that the apoptotic cell is not extruded. It seems likely the actomyosin cable has an active role to play in apoptosis-driven buckling.

Cell shortening has also been observed in other instances of epithelial invagination. In ascidian gastrulation, Sherrard *et al.* [46] showed that apical constriction of the endodermal cells actually does not drive the invagination process; rather, a basolateral accumulation of myosin leads to apicobasal shortening of the cells and initiates the invagination. In yet another mechanism, dorsal folds in the early *Drosophila* embryo at the onset of gastrulation are initiated by a basal shifting of adherens junctions of the invaginating cells, leading to a mismatch in junction positioning with neighbouring cells that helps to drive the tissue buckling [47,48]. Although it has been shown that the positions of the adherens junctions are regulated by the polarity proteins Par1 and Bazooka, the physical mechanism remains to be investigated.

## 6. Basal wedging

**Figure 5.**
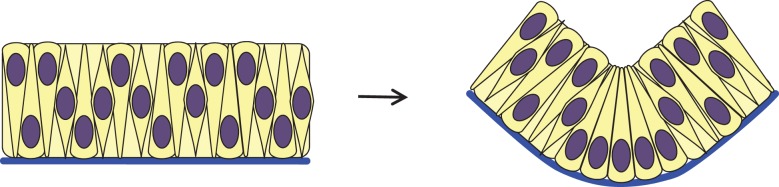
Basal wedging. Basal wedging occurs in the medial and dorsolateral hinge points of the neural plate during neural tube closure. Cells in the pseudostratified neural plate are tightly packed and only bulge around the nucleus, which moves in an apicobasal direction as the cell cycle progresses and resides basally in S-phase. Cells at the hinge point remain in S-phase longer than their neighbours, therefore becoming wedge-shaped with basal nuclei, resulting in a fold at the hinge point. Blue, basal lamina; purple, nucleus.

Wedge-shaped cells in an invaginating tissue are an inevitable consequence of the tissue geometry and do not necessarily indicate apical constriction. During neural tube development, a process called basal wedging comes into play in which wedging occurs that is quite distinct from apical constriction. At the midline of much of the forming amniote neural tube, the epithelium bends sharply to form what is known as the median hinge point (MHP) [49–51]. Cells at these hinge positions are almost all wedge-shaped, whereas their neighbours are a mixture of shapes, mostly spindle-shaped, reflecting the pseudostratified nature of this epithelium (figure 5). Importantly, the cells are very tightly packed in the plane of the epithelium, and are so narrow that each cell bulges around its nucleus. The wedge shape of hinge point cells is, at least substantially, a result of basally located nuclei. This seems to be related to interkinetic nuclear migration, which is the apicobasal movement of the nucleus as the cell cycle progresses: cells divide apically and when in S-phase the nucleus resides basally [52,53] and, consistent with this, cells at the hinge spend longer in S-phase [49,54]. The cell-division cycle has been similarly implicated in bending morphogenesis of the optic cup [55]. However, whether cell cycle control is the necessary or sole driver of apicobasal nuclear position remains an open question [56–60]. Importantly, basal wedging was experimentally distinguished from apical constriction by the finding that inhibiting actin polymerization, while causing most of the neural tube to flop open and apical surfaces to expand across the entire neural plate [41], failed to abolish bending at the median hinge point [61,62]. This also shows that median hinge bending is intrinsic, as the relaxation of the flanking epithelium uncouples the median hinge from extrinsic forces and that basal wedging occurs differently from apical constriction.

## 7. Vertical telescoping and apical/basal bunching

Intriguingly, in certain anteroposterior regions of the neural tube there are also dorsolateral hinge points that involve neither basal wedging nor (cytochalasin-sensitive) apical constriction [62]. Although extrinsic pushing force from the flanking ectoderm has been suggested as a bending mechanism [63], more recent evidence has argued against it [64] and suggested that differential cell packing generated by cell proliferation and translocation in the mouse neural tube leads to the folding of the structure [65].

Related to this, in 1986 Jacobson, Oster *et al.* [66] described in *Xenopus* frogs a cellular behaviour for neural fold elevation (the lateral beginning of neurulation) which they named ‘tractoring’. The term ‘tractoring’ was picked up and used again in the context of epithelial bending in sea urchin gastrulation in two further papers [67,68]. What these three papers address is worth considering in detail (see next paragraph). Unfortunately, the term ‘tractoring’ was also used in the same 1986 paper to describe not only the cell behaviours as such but also a speculative subcellular mechanism that could drive them. In this speculative use of the term ‘tractoring’, the cell cortex flows like a caterpillar track around the cell to move the cell relative to its neighbours [66]. It is hard to imagine cortical tractoring in epithelia with tight junctions, which would prevent cortical movement, and the idea has never been followed up (although embryonic epithelia, especially in mammalian embryos, often lack tight junctions and may have more labile adhesion). A recent paper has revived the idea of cortical tractoring for isolated cells migrating in confined spaces [69]. To avoid confusion, we will abandon the term ‘tractoring’ altogether (except in quotation marks, where those authors used it). Instead we offer two new terms—for indeed there are two cell behaviours involved—namely vertical telescoping and basal (or apical) bunching.

**Figure 6.**
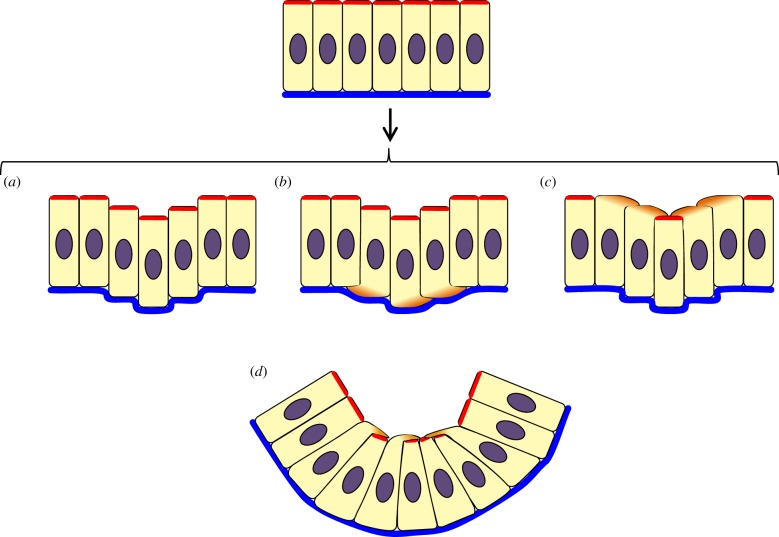
Other mechanisms; vertical telescoping and apical bunching. (*a*) In vertical telescoping the vertical shear between neighbouring cells moves cells relative to one another. (*b*) Vertical telescoping could be assisted by basal protrusions pushing neighbouring cells upwards. (*c*) Vertical telescoping could alternatively be assisted by apical protrusions pushing down on neighbouring cells. (*d*) In bunching, cells send apical or basal processes over adjacent cells, exerting lateral force to squeeze neighbouring cells and buckle the epithelial sheet. Red, actomyosin; blue, basal lamina; orange, cell protrusions; purple, nucleus.

An effect described by Jacobsen *et al.* [66] as occurring during neural plate bending was that the cells slide vertically past one another, similarly to the way that the steps of a rising escalator do, to create a slope or bend. Another useful way of describing this is that the epithelium extends downwards by vertical displacement, effectively shear, between its cells organized around the centre of the invagination, much in the way that a telescope extends by the sliding of its sections (figure 6*a*). We suggest ‘vertical telescoping’ as a term for this process to capture the idea not only of vertical ‘shear’ but also its concentric arrangement. Actual shear between cells is unlikely: the vertical cell movement is much more likely to resemble classical cell migration, in which cells crawl or roll over fixed adhesion points, with movement being effected by the extension of basal or apical protrusions (figure 6*b*,*c*). We have some preliminary evidence for vertical telescoping occurring in morphogenesis of teeth and salivary gland invagination (E. Panousopoulou, J.Li and J.B.A. Green 2016, unpublished data). The observations in the mouse lateral neural tube mentioned above [65] are consistent with this type of mechanism, but vertical shear-like movement remains to be observed directly.

A different mechanism that has been described by the term ‘tractoring’ is in sea urchin gastrulation and consists of apical protrusions of cells ‘dragging’ themselves centripetally, forcing the cells into centripetal-leaning orientations and consequentially bending the epithelium (figure 6*d*) [67]. This process is most explicitly modelled as contractile apical cell extensions in a second paper that uses the term ‘tractoring’ [68], and we here rename this process as ‘apical bunching’ (figure 6*d*), with the word ‘bunching’ conveying the idea of gathering together (of cell apices) by squeezing from the outside (by neighbouring cells' apical protrusions extended laterally). Apical bunching differs from vertical telescoping in that bunching drives shape change without vertical displacement, whereas vertical telescoping is conversely defined as vertical shear without shape change. However, these definitions are theoretical: in practice, lateral crawling of apical protrusions could simultaneously both deform and depress neighbouring cells (figure 6*d*). Apical bunching also differs from apical constriction because in bunching, force is extrinsic to the deformed cell, whereas in constriction, it is intrinsic.

Jacobson *et al.* [66] had also suggested basal protrusions of cells in the neural plate advanced laterally along the basal lamina, reaching underneath their neighbours. One effect of this appears to be to laterally compress these cells at their bases, driving the neural fold to evaginate (creating a concave invagination-like bend in the adjacent part of the neural plate passively). This could be described as ‘basal bunching’ as opposed to apical bunching, yet there are still no clear live observations of this phenomenon experimentally to confirm its existence.

## 8. Suprabasal intercalation: bending a multilayered epithelium

Most of the above mechanisms concern either monolayers or pseudostratified epithelia; therefore one remaining mystery is how a stratified epithelium, which very often appears in early organogenesis, such as in tooth placode, hair follicle and mammary gland, bends into a bud or tube-shaped organ primordium. A recent study showed that, in these bending epithelia, actin and phosphorylated myosin are not enriched apically in the wedge-shaped basal layer cells, and nuclei are not predominantly basally located [70]. Hence, neither apical constriction nor basal wedging seem to be involved in this process.

**Figure 7.**
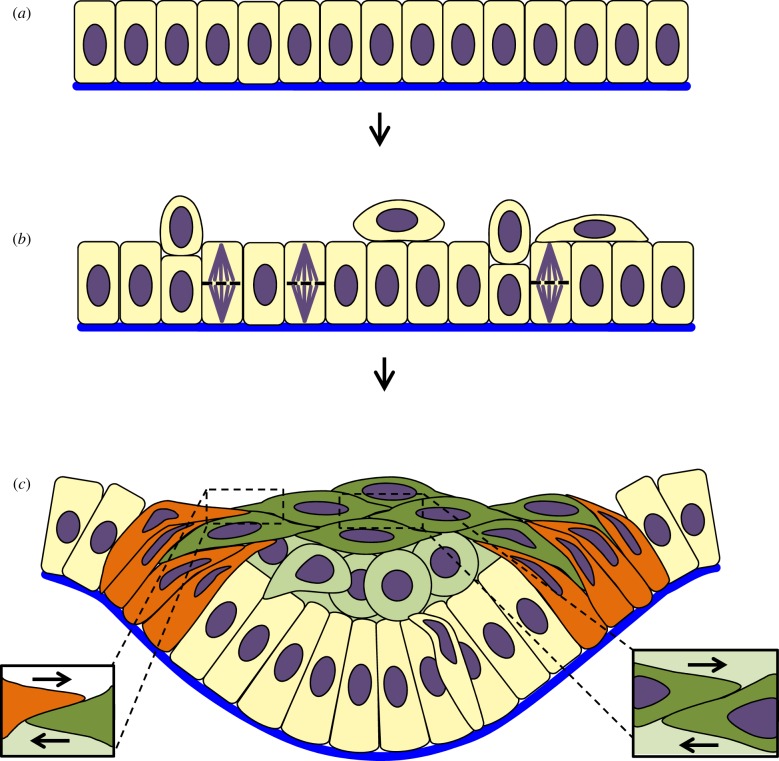
Suprabasal intercalation. (*a*) Flat epithelial monolayer with slightly columnar cells. (*b*) Cell division leads to a thickening of the epithelium, creating a placode. (*c*) Cells at the edges of the placode (orange) bend inwards and intercalate with more central cells, creating tension which leads to bending; stratification creates suprabasal cells (pale and dark green), some of which intercalate (dark green cells), creating further tension to fully bend the epithelium; boxes to the right show intercalating cells; arrows indicate the direction of cell movement. Blue, basal lamina; green, suprabasal cells; orange, shoulder cells; purple, nucleus/mitotic spindle.

Theoretically, locally elevated proliferation, and more specifically stratification, of cells above the basal layer has been proposed to be sufficient to drive ‘down growth’ of an epithelium (figure 7) [71]; indeed, examination of spindle orientation in the molar tooth, one of the largest epithelial organ placodes, showed that cell division in the placode occurs perpendicular to the plane of the tissue, creating the suprabasal cells (figure 7*b*) [72]. However, *a priori*, stratification would be expected to thicken an epithelium both upwards and downwards, or even just upwards if the underlying (mesenchymal) tissue were stiff. Moreover, experimentally, it was also discovered in the same piece of work that stratification alone is not enough to drive invagination and inhibition of proliferation does not inhibit invagination [72]. In other words, ‘down growth’ is an inadequate description for early placode invagination. Instead, suprabasal cells were found to generate the essential bending tension, as revealed by observation of elevated actin and phosphomyosin, cell intercalation movements and recoil upon physical cutting [70]. The planar tension created in suprabasal layers by planar cell intercalation was shown to be transmitted to the basal lamina by basal layer cells that are anchored basally but simultaneously extend centripetally orientated apical protrusions that participate in the intercalation (figure 7*c*) [70]. The basal layer resists lateral compression and so must bend in response to the suprabasal contraction. Topologically, suprabasal cells in the ectodermal placodes take the role of apical actomyosin cables, but on a much larger scale.

## 9. Conclusion

As specified in §1, we have here attempted to provide a brief, up-to-date summary of the main mechanisms thought to be involved in epithelial invagination. It is worth mentioning that the different mechanisms discussed here are not necessarily mutually exclusive. For example, proliferation is a necessary condition for suprabasal intercalation in stratified epithelium, basal relaxation normally precedes apical constriction, and apical or basal bunching can act together with apical constriction or basal wedging. The hierarchy of the mechanisms discussed also represents the limitations of our knowledge. Apical constriction is, perhaps, assumed to be common mostly on the basis of its obviousness in the early development of model laboratory organisms. The other mechanisms are progressively less appreciated, but deserve to be considered on a more equal footing, as they could be more common and important in later development and across diverse species than hitherto appreciated. Invagination is just one type of epithelial bending. We have omitted, for space reasons, discussion of the most obviously related morphogenetic process, namely evagination, for example by basal constriction, leading to an outward folding of tissue [73]. We have also limited this review by focusing on bending that is driven by intrinsic forces. By ‘intrinsic’ we mean forces generated within an epithelium itself (although not necessarily just at the bending point, as exemplified by cable-driven buckling). Beside the intrinsic forces, bending of tubes such as the gut or heart can be driven by forces extrinsic to the epithelium, such as resistive forces generated in attached or enclosing inelastic tissue as the epithelium itself grows [74–77].

Rather than focus, for example, on biomechanical aspects of epithelial bending [1,2] or comprehensively review epithelial morphogenesis as a whole [3], we have provided a sketch of a variety of cell systems that by coordinated ensemble behaviours generate the required anatomy. For some of these, there is some understanding of molecular mechanisms, but for most, the connection between subcellular molecular processes and supracellular tissue-level outcomes remains crude. However, what is clear is that it is illuminating to consider the mechanism at a supracellular or multicellular scale. By considering epithelial invaginations in this way as systems of cells, the dazzling variety of developmental events may be reducible to a small number of tractable motifs. Identifying and characterizing these motifs (even with variations) thus becomes a feasible agenda for both experimental and theoretical progress.

## References

[1] Davidson LA 2012 Epithelial machines that shape the embryo. Trends Cell Biol. 22, 82–87. (10.1016/j.tcb.2011.10.005) 22130222 PMC3273669

[2] Ettensohn CA 1985 Mechanisms of epithelial invagination. Q. Rev. Biol. 60, 289–307. (10.1086/414426) 3901078

[3] Fristrom D 1988 The cellular basis of epithelial morphogenesis. A review. Tissue Cell 20, 645–690. (10.1016/0040-8166(88)90015-8) 3068832

[4] Sawyer JM, Harrell JR, Shemer G, Sullivan-Brown J, Roh-Johnson M, Goldstein B 2010 Apical constriction: a cell shape change that can drive morphogenesis. Dev. Biol. 341, 5–19. (10.1016/j.ydbio.2009.09.009) 19751720 PMC2875788

[5] Polyakov O, He B, Swan M, Shaevitz JW, Kaschube M, Wieschaus E 2014 Passive mechanical forces control cell-shape change during *Drosophila* ventral furrow formation. Biophys. J. 107, 998–1010. (10.1016/j.bpj.2014.07.013) 25140436 PMC4142243

[6] Davidson LA 2012 No strings attached: new insights into epithelial morphogenesis. BMC Biol. 10, 105 (10.1186/1741-7007-10-105) 23256891 PMC3527222

[7] Keller R, Shook D 2011 The bending of cell sheets—from folding to rolling. BMC Biol. 9, 90 (10.1186/1741-7007-9-90) 22206439 PMC3248374

[8] Kondo T, Hayashi S 2015 Mechanisms of cell height changes that mediate epithelial invagination. Dev. Growth Differ. 57, 313–323. (10.1111/dgd.12224) 25988719

[9] Martin AC, Goldstein B 2014 Apical constriction: themes and variations on a cellular mechanism driving morphogenesis. Development 141, 1987–1998. (10.1242/dev.102228) 24803648 PMC4011084

[10] St Johnston D, Sanson B 2011 Epithelial polarity and morphogenesis. Curr. Opin. Cell Biol. 23, 540–546. (10.1016/j.ceb.2011.07.005) 21807488

[11] Lewis WH 1947 Mechanics of invagination. Anat. Rec. 97, 139–156. (10.1002/ar.1090970203) 20284907

[12] Baker PC, Schroede TE 1967 Cytoplasmic filaments and morphogenetic movement in amphibian neural tube. Dev. Biol. 15, 432–450. (10.1016/0012-1606(67)90036-X) 6032487

[13] Lang RA, Herman K, Reynolds AB, Hildebrand JD, Plageman TF Jr 2014 p120-catenin-dependent junctional recruitment of Shroom3 is required for apical constriction during lens pit morphogenesis. Development 141, 3177–3187. (10.1242/dev.107433) 25038041 PMC4197547

[14] Lee JY, Harland RM 2007 Actomyosin contractility and microtubules drive apical constriction in *Xenopus* bottle cells. Dev. Biol. 311, 40–52. (10.1016/j.ydbio.2007.08.010) 17868669 PMC2744900

[15] Martin AC, Kaschube M, Wieschaus EF 2009 Pulsed contractions of an actin-myosin network drive apical constriction. Nature 457, 495–499. (10.1038/nature07522) 19029882 PMC2822715

[16] Mason FM, Tworoger M, Martin AC 2013 Apical domain polarization localizes actin-myosin activity to drive ratchet-like apical constriction. Nat. Cell Biol. 15, 926–936. (10.1038/ncb2796) 23831726 PMC3736338

[17] Borges RM, Lamers ML, Forti FL, Santos MF, Yan CY 2011 Rho signaling pathway and apical constriction in the early lens placode. Genesis 49, 368–379. (10.1002/dvg.20723) 21309072

[18] Sai X, Yonemura S, Ladher RK 2014 Junctionally restricted RhoA activity is necessary for apical constriction during phase 2 inner ear placode invagination. Dev. Biol. 394, 206–216. (10.1016/j.ydbio.2014.08.022) 25173873

[19] Haigo SL, Hildebrand JD, Harland RM, Wallingford JB 2003 Shroom induces apical constriction and is required for hingepoint formation during neural tube closure. Curr. Biol. 13, 2125–2137. (10.1016/j.cub.2003.11.054) 14680628

[20] Hildebrand JD 2005 Shroom regulates epithelial cell shape via the apical positioning of an actomyosin network. J. Cell Sci. 118, 5191–5203. (10.1242/jcs.02626) 16249236

[21] Hildebrand JD, Soriano P 1999 Shroom, a PDZ domain-containing actin-binding protein, is required for neural tube morphogenesis in mice. Cell 99, 485–497. (10.1016/S0092-8674(00)81537-8) 10589677

[22] Plageman TF Jr, Chung MI, Lou M, Smith AN, Hildebrand JD, Wallingford JB, Lang RA 2010 Pax6-dependent Shroom3 expression regulates apical constriction during lens placode invagination. Development 137, 405–415. (10.1242/dev.045369) 20081189 PMC2858910

[23] Jidigam VK, Srinivasan RC, Patthey C, Gunhaga L 2015 Apical constriction and epithelial invagination are regulated by BMP activity. Biol Open 4, 1782–1791. (10.1242/bio.015263) 26621830 PMC4736041

[24] Martin AC, Gelbart M, Fernandez-Gonzalez R, Kaschube M, Wieschaus EF 2010 Integration of contractile forces during tissue invagination. J. Cell Biol. 188, 735–749. (10.1083/jcb.200910099) 20194639 PMC2835944

[25] Maître J-L, Niwayama R, Turlier H, Nédélec F, Hiiragi T 2015 Pulsatile cell-autonomous contractility drives compaction in the mouse embryo. Nat. Cell Biol. 17, 849–855. (10.1038/ncb3185) 26075357

[26] Samarage CR, White MD, Álvarez YD, Fierro-González JC, Henon Y, Jesudason EC, Bissiere S, Fouras A, Plachta N 2015 Cortical tension allocates the first inner cells of the mammalian embryo. Dev. Cell 34, 435–447. (10.1016/j.devcel.2015.07.004) 26279486

[27] He B, Doubrovinski K, Polyakov O, Wieschaus E 2014 Apical constriction drives tissue-scale hydrodynamic flow to mediate cell elongation. Nature 508, 392–396. (10.1038/nature13070) 24590071 PMC4111109

[28] Andrew DJ, Henderson KD, Seshaiah P 2000 Salivary gland development in *Drosophila* *melanogaster*. Mech. Dev. 92, 5–17. (10.1016/S0925-4773(99)00321-4) 10704884

[29] Kerman BE, Cheshire AM, Andrew DJ 2006 From fate to function: the *Drosophila* trachea and salivary gland as models for tubulogenesis. Differentiation 74, 326–348. (10.1111/j.1432-0436.2006.00095.x) 16916373 PMC2827874

[30] Monier B, Gettings M, Gay G, Mangeat T, Schott S, Guarner A, Suzanne M 2015 Apico-basal forces exerted by apoptotic cells drive epithelium folding. Nature 518, U245–U252. (10.1038/nature14152) 25607361

[31] Sai X, Ladher RK 2008 FGF signaling regulates cytoskeletal remodeling during epithelial morphogenesis. Curr. Biol. 18, 976–981. (10.1016/j.cub.2008.05.049) 18583133

[32] Sai X, Ladher RK 2015 Early steps in inner ear development: induction and morphogenesis of the otic placode. Front. Pharmacol. 6, 19 (10.3389/fphar.2015.00019) 25713536 PMC4322616

[33] Lomakin AJ, Lee KC, Han SJ, Bui DA, Davidson M, Mogilner A, Danuser G 2015 Competition for actin between two distinct F-actin networks defines a bistable switch for cell polarization. Nat. Cell Biol. 17, 1435–1445. (10.1038/ncb3246) 26414403 PMC4628555

[34] Roper K 2012 Anisotropy of Crumbs and aPKC drives myosin cable assembly during tube formation. Dev. Cell 23, 939–953. (10.1016/j.devcel.2012.09.013) 23153493 PMC3562440

[35] Roper K 2013 Supracellular actomyosin assemblies during development. Bioarchitecture 3, 45–49. (10.4161/bioa.25339) 23760352 PMC3715543

[36] Nishimura M, Inoue Y, Hayashi S 2007 A wave of EGFR signaling determines cell alignment and intercalation in the *Drosophila* tracheal placode. Development 134, 4273–4282. (10.1242/dev.010397) 17978004

[37] Nishimura T, Takeichi M 2008 Shroom3-mediated recruitment of Rho kinases to the apical cell junctions regulates epithelial and neuroepithelial planar remodeling. Development 135, 1493–1502. (10.1242/dev.019646) 18339671

[38] Fernandez-Gonzalez R, Simoes Sde M, Roper JC, Eaton S, Zallen JA 2009 Myosin II dynamics are regulated by tension in intercalating cells. Dev. Cell 17, 736–743. (10.1016/j.devcel.2009.09.003) 19879198 PMC2854079

[39] Franke JD, Montague RA, Kiehart DP 2005 Nonmuscle myosin II generates forces that transmit tension and drive contraction in multiple tissues during dorsal closure. Curr. Biol. 15, 2208–2221. (10.1016/j.cub.2005.11.064) 16360683

[40] Solon J, Kaya-Copur A, Colombelli J, Brunner D 2009 Pulsed forces timed by a ratchet-like mechanism drive directed tissue movement during dorsal closure. Cell 137, 1331–1342. (10.1016/j.cell.2009.03.050) 19563762

[41] Nishimura T, Honda H, Takeichi M 2012 Planar cell polarity links axes of spatial dynamics in neural-tube closure. Cell 149, 1084–1097. (10.1016/j.cell.2012.04.021) 22632972

[42] Kondo T, Hayashi S 2013 Mitotic cell rounding accelerates epithelial invagination. Nature 494, 125–129. (10.1038/nature11792) 23334416

[43] Kiehart DP 2015 Epithelial morphogenesis: apoptotic forces drive cell shape changes. Dev. Cell 32, 532–533. (10.1016/j.devcel.2015.02.020) 25758861 PMC4494740

[44] Monier B, Suzanne M 2015 The morphogenetic role of apoptosis. Curr. Top. Dev. Biol. 114, 335–362. (10.1016/bs.ctdb.2015.07.027) 26431573

[45] Manjon C, Sanchez-Herrero E, Suzanne M 2007 Sharp boundaries of Dpp signalling trigger local cell death required for *Drosophila* leg morphogenesis. Nat. Cell Biol. 9, 57–63. (10.1038/ncb1518) 17143268

[46] Sherrard K, Robin F, Lemaire P, Munro E 2010 Sequential activation of apical and basolateral contractility drives ascidian endoderm invagination. Curr. Biol. 20, 1499–1510. (10.1016/j.cub.2010.06.075) 20691592 PMC4088275

[47] Wang YC, Khan Z, Kaschube M, Wieschaus EF 2012 Differential positioning of adherens junctions is associated with initiation of epithelial folding. Nature 484, 390–393. (10.1038/nature10938) 22456706 PMC3597240

[48] Wang YC, Khan Z, Wieschaus EF 2013 Distinct Rap1 activity states control the extent of epithelial invagination via alpha-catenin. Dev. Cell 25, 299–309. (10.1016/j.devcel.2013.04.002) 23623612 PMC3741050

[49] Smith JL, Schoenwolf GC 1987 Cell cycle and neuroepithelial cell shape during bending of the chick neural plate. Anat. Rec. 218, 196–206. (10.1002/ar.1092180215) 3619087

[50] Smith JL, Schoenwolf GC, Quan J 1994 Quantitative analyses of neuroepithelial cell shapes during bending of the mouse neural plate. J. Comp. Neurol. 342, 144–151. (10.1002/cne.903420113) 8207124

[51] Schoenwolf GC, Franks MV 1984 Quantitative analyses of changes in cell shapes during bending of the avian neural plate. Dev. Biol. 105, 257–272. (10.1016/0012-1606(84)90284-7) 6479439

[52] Spear PC, Erickson CA 2012 Apical movement during interkinetic nuclear migration is a two-step process. Dev. Biol. 370, 33–41. (10.1016/j.ydbio.2012.06.031) 22884563 PMC3935435

[53] Spear PC, Erickson CA 2012 Interkinetic nuclear migration: a mysterious process in search of a function. Dev. Growth Differ. 54, 306–316. (10.1111/j.1440-169X.2012.01342.x) 22524603 PMC3357188

[54] Smith JL, Schoenwolf GC 1988 Role of cell-cycle in regulating neuroepithelial cell shape during bending of the chick neural plate. Cell Tissue Res. 252, 491–500. (10.1007/BF00216636) 3396052

[55] Eiraku M, Takata N, Ishibashi H, Kawada M, Sakakura E, Okuda S, Sekiguchi K, Adachi T, Sasai Y 2011 Self-organizing optic-cup morphogenesis in three-dimensional culture. Nature 472, 51–U73. (10.1038/nature09941) 21475194

[56] Guthrie S, Butcher M, Lumsden A 1991 Patterns of cell division and interkinetic nuclear migration in the chick embryo hindbrain. J. Neurobiol. 22, 742–754. (10.1002/neu.480220709) 1722508

[57] Kosodo Y, Suetsugu T, Suda M, Mimori-Kiyosue Y, Toida K, Baba SA, Kimura A, Matsuzaki F 2011 Regulation of interkinetic nuclear migration by cell cycle-coupled active and passive mechanisms in the developing brain. EMBO J. 30, 1690–1704. (10.1038/emboj.2011.81) 21441895 PMC3101991

[58] Norden C, Young S, Link BA, Harris WA 2009 Actomyosin is the main driver of interkinetic nuclear migration in the retina. Cell 138, 1195–1208. (10.1016/j.cell.2009.06.032) 19766571 PMC2791877

[59] Schenk J, Wilsch-Brauninger M, Calegari F, Huttner WB 2009 Myosin II is required for interkinetic nuclear migration of neural progenitors. Proc. Natl Acad. Sci. USA 106, 16 487–16 492. (10.1073/pnas.0908928106) PMC2752599 19805325

[60] Tsutsumi Y, Fushiki S 2000 Comparison of cell kinetics between the boundary and the interboundary areas during hindbrain segmentation in the chick embryo. Acta Histochem. Cytochem. 33, 141–147. (10.1267/ahc.33.141)

[61] Schoenwolf GC, Folsom D, Moe A 1988 A reexamination of the role of microfilaments in neurulation in the chick embryo. Anat. Rec. 220, 87–102. (10.1002/ar.1092200111) 3348489

[62] Ybot-Gonzalez P, Copp AJ 1999 Bending of the neural plate during mouse spinal neurulation is independent of actin microfilaments. Dev. Dyn. 215, 273–283. (10.1002/(SICI)1097-0177(199907)215:3%3C273::AID-AJA9%3E3.0.CO;2-H) 10398537

[63] Alvarez IS, Schoenwolf GC 1992 Expansion of surface epithelium provides the major extrinsic force for bending of the neural plate. J. Exp. Zool. 261, 340–348. (10.1002/jez.1402610313) 1629665

[64] Ybot-Gonzalez P, Cogram P, Gerrelli D, Copp AJ 2002 Sonic hedgehog and the molecular regulation of mouse neural tube closure. Development 129, 2507–2517. 11973281 10.1242/dev.129.10.2507

[65] McShane SG, Mole MA, Savery D, Greene NDE, Tam PPL, Copp AJ 2015 Cellular basis of neuroepithelial bending during mouse spinal neural tube closure. Dev. Biol. 404, 113–124. (10.1016/j.ydbio.2015.06.003) 26079577 PMC4528075

[66] Jacobson AG, Oster GF, Odell GM, Cheng LY 1986 Neurulation and the cortical tractor model for epithelial folding. J. Embryol. Exp. Morphol. 96, 19–49. 3805983

[67] Burke RD, Myers RL, Sexton TL, Jackson C 1991 Cell movements during the initial phase of gastrulation in the sea urchin embryo. Dev. Biol. 146, 542–557. (10.1016/0012-1606(91)90255-2) 1864470

[68] Davidson LA, Koehl MA, Keller R, Oster GF 1995 How do sea urchins invaginate? Using biomechanics to distinguish between mechanisms of primary invagination. Development 121, 2005–2018. 7635048 10.1242/dev.121.7.2005

[69] Bergert M, Erzberger A, Desai RA, Aspalter IM, Oates AC, Charras G, Salbreux G, Paluch EK 2015 Force transmission during adhesion-independent migration. Nat. Cell Biol. 17, 524–529. (10.1038/ncb3134) 25774834 PMC6485532

[70] Panousopoulou E, Green JB 2016 Invagination of ectodermal placodes is driven by cell intercalation-mediated contraction of the suprabasal tissue canopy. PLoS Biol. 14, e1002405 (10.1371/journal.pbio.1002405) 26960155 PMC4784948

[71] Basan M, Joanny JF, Prost J, Risler T 2011 Undulation instability of epithelial tissues. Phys. Rev. Lett. 106, 158101 (10.1103/PhysRevLett.106.158101) 21568616

[72] Li J, Chatzeli L, Panousopoulou E, Tucker AS, Green JB 2016 Epithelial stratification and placode invagination are separable functions in early morphogenesis of the molar tooth. Development 143, 670–681. (10.1242/dev.130187) 26755699 PMC4760321

[73] Gutzman JH, Graeden EG, Lowery LA, Holley HS, Sive H 2008 Formation of the zebrafish midbrain-hindbrain boundary constriction requires laminin-dependent basal constriction. Mech. Dev. 125, 974–983. (10.1016/j.mod.2008.07.004) 18682291 PMC2780020

[74] Savin T, Kurpios NA, Shyer AE, Florescu P, Liang H, Mahadevan L, Tabin CJ 2011 On the growth and form of the gut. Nature 476, 57–62. (10.1038/nature10277) 21814276 PMC3335276

[75] Shyer AE, Tallinen T, Nerurkar NL, Wei Z, Gil ES, Kaplan DL, Tabin CJ, Mahadevan L 2013 Villification: how the gut gets its villi. Science 342, 212–218. (10.1126/science.1238842) 23989955 PMC4045245

[76] Voronov DA, Alford PW, Xu G, Taber LA 2004 The role of mechanical forces in dextral rotation during cardiac looping in the chick embryo. Dev. Biol. 272, 339–350. (10.1016/j.ydbio.2004.04.033) 15282152

[77] Voronov DA, Taber LA 2002 Cardiac looping in experimental conditions: effects of extraembryonic forces. Dev. Dyn. 224, 413–421. (10.1002/dvdy.10121) 12203733

